# Impact of Gamification on the Self-Efficacy and Motivation to Quit of Smokers: Observational Study of Two Gamified Smoking Cessation Mobile Apps

**DOI:** 10.2196/27290

**Published:** 2021-04-27

**Authors:** Nikita B Rajani, Nikolaos Mastellos, Filippos T Filippidis

**Affiliations:** 1 Department of Primary Care and Public Health Imperial College London London United Kingdom

**Keywords:** gamification, smoking cessation, mobile applications, self-efficacy, motivation to quit, mHealth, mobile phone

## Abstract

**Background:**

The proportion of smokers making quit attempts and the proportion of smokers successfully quitting have been decreasing over the past few years. Previous studies have shown that smokers with high self-efficacy and motivation to quit have an increased likelihood of quitting and staying quit. Consequently, further research on strategies that can improve the self-efficacy and motivation of smokers seeking to quit could lead to substantially higher cessation rates. Some studies have found that gamification can positively impact the cognitive components of behavioral change, including self-efficacy and motivation. However, the impact of gamification in the context of smoking cessation and mobile health has been sparsely investigated.

**Objective:**

This study aims to examine the association between perceived usefulness, perceived ease of use, and frequency of use of gamification features embedded in smoking cessation apps on self-efficacy and motivation to quit smoking.

**Methods:**

Participants were assigned to use 1 of the 2 mobile apps for a duration of 4 weeks. App-based questionnaires were provided to participants before app use and 2 weeks and 4 weeks after they started using the app. Gamification was quantitatively operationalized based on the Cugelman gamification framework and concepts from the technology acceptance model. The mean values of perceived frequency, ease of use, and usefulness of gamification features were calculated at midstudy and end-study. Two linear regression models were used to investigate the impact of gamification on self-efficacy and motivation to quit.

**Results:**

A total of 116 participants completed the study. The mean self-efficacy increased from 37.38 (SD 13.3) to 42.47 (SD 11.5) points and motivation to quit increased from 5.94 (SD 1.4) to 6.32 (SD 1.7) points after app use. *Goal setting* was perceived to be the most useful gamification feature, whereas *sharing* was perceived to be the least useful. Participants self-reported that they used the progress dashboards the most often, whereas they used the sharing feature the least often. The average perceived frequency of gamification features was statistically significantly associated with change in self-efficacy (*β*=3.35; 95% CI 0.31-6.40) and change in motivation to quit (*β*=.54; 95% CI 0.15-0.94) between baseline and end-study.

**Conclusions:**

Gamification embedded in mobile apps can have positive effects on self-efficacy and motivation to quit smoking. The findings of this study can provide important insights for tobacco control policy makers, mobile app developers, and smokers seeking to quit.

## Introduction

Smoking is the second leading risk factor for early death and disability worldwide, with approximately 8 million deaths annually [1,2]. In the United Kingdom, 16% of all deaths in 2016 were attributed to smoking [3]. Despite most smokers wanting to quit, research shows that the number of smokers in the United Kingdom who tried to quit in the past year dropped by 7% between 2008 and 2017 [4]. Among the smokers making attempts, only 3%-5% are successful in staying quit after a year [5]. Exploring methods that can improve the success rates of long-term quitting can lead to a decreased prevalence of chronic diseases and premature mortality. Previous studies have shown that high motivation to quit and self-efficacy, 2 factors often integrated into face-to-face behavioral support interventions, have been found to increase the likelihood of attempting to quit and successfully quitting [6-9].

Motivation to quit refers to the level of determination and importance placed by an individual on quitting smoking [10]. On the other hand, self-efficacy, a theoretical construct first termed by Bandura [11], in the context of smoking cessation refers to one’s confidence in their ability to refrain from smoking when faced with internal and external stimuli [11,12]. Several strategies are adopted by health behavior change interventions to influence self-efficacy or motivation to quit. For example, motivational interviewing is often used to help smokers explore reasons for quitting and “make them feel more willing and able to stop smoking” [13]. Similarly, vicarious experience and performance attainment are strategies to influence self-efficacy. Vicarious experience involves “exposing the individual to successful behaviour performances or gaining experience through practice” [14]. In the context of smoking cessation, this could mean showing smokers examples of other smokers who successfully quit after attending cessation programs. Performance attainment refers to having successful experiences [11,14]; for a smoker trying to quit, this could mean staying abstinent for a day and recognizing this as a success. Such strategies have been integrated into both face-to-face and digital interventions, such as mobile health (mHealth) solutions, which have become increasingly important to improve access, knowledge, and behavior across different contexts and population groups [15].

One strategy that has been frequently applied across both physical and remote interventions for behavioral change is gamification, also known as the use of game elements in a nongame context [16]. Some examples of game elements include achievement badges, goal setting, progress tracking, sharing progress, and levels [17]. Past studies have found that gamification can positively impact cognitive components of behavioral change, including self-efficacy and motivation. For example, self-efficacy was the most cited advantage of gamified classrooms, as it improved the confidence levels of students [18]. Similarly, Thorsteinsen et al [19] found that gaming elements significantly increased the motivation of individuals to engage in physical activity. The use of gamification has gradually become more popular as it appears to share key components with several behavioral change theories and techniques [17,20]. For example, self-determination theory, a dominant theory of motivation, suggests that gamification elements such as points and badges serve as informational feedback instilling a sense of intrinsically motivating competence in the user [21]. Similarly, goal-setting theory, another prominent theory of motivation, has been associated with gamification; for example, elements such as leaderboards and levels provide individuals with smaller, more immediate goals that can improve task performance and, in turn, increase one’s confidence in their ability to complete tasks [21,22].

Although gamification seems promising, the impact of gamification in the field of health behavior change has focused primarily on improving physical activity levels [23-25]. More specifically, there is room to further study the use of gamification in the context of other behaviors, such as smoking. With the proliferation of smartphone use and the increased availability of digitalized interventions, it is vital to investigate novel strategies, such as gamification, that complement mHealth solutions and are in line with current digitalization trends. The majority of studies that have explored the impact of gamification in the context of smoking cessation have been purely qualitative and consequently have not operationalized gamification quantitatively. For example, Pløhn and Aalberg [26] interviewed participants after they used a digital smoking cessation intervention with gamification features and found positive perceptions of gamification as an important motivational factor to aid quitting. However, the smoking cessation intervention was not delivered via a mobile app. Another study by El-Hilly et al [27] found promising results on the effect of gamification on the motivation and engagement of smokers within the context of mHealth. Similar to the study by Pløhn and Aalberg [26], the study by El-Hilly et al [27] was also qualitative and had a small sample size (n=16), hindering the external validity of the findings. On the other hand, Lin et al [28] investigated gamification quantitatively and found that program progress or step completion as a gamification element in a smoking cessation mobile app had a positive impact on psychological factors such as user well-being, inspiration, and empowerment [28]. It would be worthwhile to investigate whether gamification in smoking cessation apps can also positively impact essential cognitive factors such as self-efficacy and motivation to quit.

Exploring whether gamification in the context of mHealth can increase the self-efficacy and motivation of smokers could lead to the design of more tailored interventions, which, in turn, could improve cessation rates and reduce the health burden of tobacco consumption. The findings of this study could also provide insights into the effective design of mobile apps. Moreover, because of the wide reach and low cost of mHealth solutions, understanding the effects of gamification on mHealth could have considerable effects on helping disadvantaged groups and reducing health inequalities [29]. On the basis of the limitations of prior research, this study’s aim of exploring gamified smoking cessation apps can also help enhance the current understanding of the effectiveness of mHealth interventions for behavior change and extend our knowledge of novel methods to promote healthy lifestyle changes. Specifically, our study aims to quantitatively assess the association between overall perceived usefulness, ease of use, and frequency of use of gamification features and the self-efficacy and motivation of smokers seeking to quit. 

## Methods

### Sampling and Eligibility

On the basis of an a priori analysis using a power level of 1−*β*=.80 and a significance level α=.05, we aimed to recruit 140 participants. The results of a previous study examining the impact of gamification elements in a fitness app on perceived competence, a proxy for self-efficacy, indicated that 112 participants were required to complete the study [25]. Similar to previous mobile app studies, we considered a dropout rate of approximately 20% [30-32], which resulted in the recruitment of 140 participants. The sample size calculation also assumes that both apps are similar based on a prior mobile app review [33].

Participants were required to be at least 18 years old and current smokers (at least one cigarette a day and 100 cigarettes smoked in their lifetime) to be eligible. Moreover, to take part in the study, individuals had to report that they were trying or willing to quit smoking in the next 30 days and were not using other forms of smoking cessation treatments. Individuals diagnosed with mental health conditions were excluded from the study.

### Study Design

A 4-week observational study assessing the association between gamification, self-efficacy, and motivation to quit was conducted from June 2019 to July 2020. No face-to-face contact was required, and the study was conducted on the internet. Participants were recruited via social media, and posters were displayed in public places in London. Initially, participants who expressed interest in the study (N=326) were screened to assess their eligibility. Eligible participants provided informed consent (n=170) and were assigned a participant identification number (PID). They were then requested to complete a baseline questionnaire that asked about general demographics (age, gender, education, marital status, education, country of residence, etc), smoking habits (number of cigarettes smoked, nicotine dependence, etc), self-efficacy, and motivation to quit.

In total, 154 participants completed the baseline assessment and were provided instructions on how to download and start using the app. Even-numbered PIDs were assigned to the mobile app Quit Genius, and odd-numbered PIDs were assigned to the mobile app Kwit. This deterministic method was used to ensure an equal split of participants between the 2 apps. Participants were asked to use the assigned mobile app for a total of 4 weeks. A midstudy questionnaire after 2 weeks of using the app and an end-study questionnaire after 4 weeks of using the app were given to participants.

Of those participants who completed the baseline assessment, 138 installed the app and 116 completed all 4 weeks of the study. Midstudy and end-study assessments included questions regarding gamification, self-efficacy, and motivation to quit. Participants were incentivized via free access to all features of the app and a chance to win a £50 (US $68) Amazon voucher. An overview of the number of participants at each stage of the study is presented in Multimedia Appendix 1.

### Mobile Apps

Mobile apps for the study were selected based on a mobile app review that found these 2 apps to have a high embedment of gamification features and a high adherence to smoking cessation guidelines in the United Kingdom [33,34]. Screenshots of both apps are shown in Multimedia Appendix 2.

#### Kwit

Kwit is a smoking cessation mobile app that helps individuals starting their quit journey and individuals trying to stay quit [35]. The app includes several features such as a calculator, a smoking diary that helps smokers log and analyze cravings and triggers, motivation cards, social media sharing, levels, and achievement cards. The app is based on cognitive behavioral therapy (CBT) and gamification strategies. The versions of Kwit used during the study period included those released from June 2019 (v.4.1) to July 2020 (v.4.4).

#### Quit Genius

Quit Genius is a gamified smoking cessation mobile app based on CBT [36]. It delivers personalized support to individuals seeking to quit smoking and helps quitters maintain their quit status. It includes several features such as a tracker, a daily diary that allows quitters to log their cravings and triggers, a quitting toolbox, a goal-setting feature, achievement badges, stages of information that build upon each other, and a quit coach who provides continuous personalized support. The versions downloaded by participants were those released from June 2019 (v.1.1) to July 2020 (v.1.9). 

### Measures

#### Sociodemographic Factors

Common sociodemographic factors were assessed at baseline. Age in years was categorized as 18-29, 30-41, 42-53, or 54-65 years, and gender was categorized as male or female. Marital status was categorized as single or married or civil partnered. Education was based on United Nations Educational, Scientific and Cultural Organization’s classification into 3 categories: low if primary school was completed, medium if secondary school was completed, and high if a college or university degree was attained [37]. Employment status was categorized as unemployed (individuals who are willing and able to work but have no employment), employed, or nonemployed (individuals who are unable to work, including students and homemakers). Residence was categorized based on the World Health Organization regions: Western Pacific, Americas, Southeast Asia, Europe, Africa, and Eastern Mediterranean [38].

#### Nicotine Dependence

The Fagerström test with 6 items was used to measure participants’ tolerance of and dependence on nicotine [36]. On the basis of the responses, participants were categorized into 3 levels: low (0-4 points), moderate (5-7 points), and high (8-10 points) [39,40].

#### Self-Efficacy

The self-efficacy of a participant was measured using a 12-item scale called The Smoking Self-Efficacy Questionnaire [12]. The scale assesses an individual’s confidence in their ability to refrain from smoking when faced with internal and external stimuli. Response options included not at all sure, not very sure, more or less sure, fairly sure, and absolutely sure. A total score ranging from 12 to 60 was computed for each participant, with higher scores signifying higher self-efficacy.

#### Motivation to Quit

Participants were asked 2 items frequently used in cessation studies to measure their motivation to quit smoking [10,41,42]. The first item asked was as follows: How important is it to you to give up smoking altogether at this attempt? Responses included the following: desperately important, very important, quite important, and not all that important. The second item asked was as follows: How determined are you to give up smoking at this attempt? Response options included the following: extremely determined, very determined, quite determined, and not all that determined. A total score ranging from 2 to 8 was calculated for each participant, with higher scores signifying higher motivation.

#### Gamification

Gamification features for each app were identified using Cugelman framework for gamification strategies and tactics and are displayed in Multimedia Appendix 3 [17]. For each identified gamification feature, participants were asked how useful and easy to use they found it during their quit attempt. Participants were provided with 5-point Likert scale responses: strongly agree, agree, neither agree nor disagree, disagree, and strongly disagree. Perceived usefulness and ease of use are 2 vital components of the technology acceptance model, which has been widely used in existing literature to better understand user acceptance and attitudes toward mobile apps and app features [43,44].

Participants were also asked how frequently they engaged with each gamification element or feature during their quit attempt. Participants were provided with 5-point Likert scale responses: almost always, often, sometimes, rarely, and never. Responses were assigned points ranging from 1 to 5 for each gamification feature. A pooled mean was calculated for all features, with a higher mean (from 1 to 5) indicating greater overall engagement with gamification.

### Statistical Analysis

The statistical software STATA 13.1 (StataCorp), was used for the analyses. Box and whisker plots were created to present an overview of self-efficacy and motivation to quit levels of participants at various study time points. The mean values of perceived frequency, ease of use, and usefulness of gamification features were calculated at midstudy and end-study. Two-way paired sample *t* tests were used to test whether differences in self-efficacy and motivation to quit at various time points of the study were statistically significant. In addition, we explored various linear regression models to examine whether gamification was associated with changes in self-efficacy and motivation to quit. On the basis of an iterative process that considered the fit of the data with our model (ie, comparing the Akaike information criterion and Bayesian information criterion), 2 linear regression models were performed. The first tested the association between perceived ease of use, frequency of use, and usefulness of gamification with change in self-efficacy, and the second tested the association between perceived ease of use, frequency of use, and usefulness of gamification with change in motivation to quit. Both models controlled for age, gender, education, marital status, nicotine dependence, baseline self-efficacy, and baseline motivation to quit. Significance at the 5% level (0.05), along with 95% CIs for all included coefficients, is presented in the *Results* section.

## Results

### Study Participants

As shown in Table 1, there was an equal split of participants who used the apps Kwit (58/116, 50%) and Quit Genius (58/116, 50%). The majority of participants were male (71/116, 61.2%), highly educated (87/116, 75%), single (77/116, 66.4%), employed (76/116, 65.6%), and living in Europe (67/116, 57.8%). More than half of the participants smoked 10 or fewer cigarettes on a daily basis (63/116, 54.3%), and the majority had low to moderate dependence on nicotine (107/116, 92.2%).

**Table 1:** Sociodemographic and general characteristics of participants (n=116).

Characteristics		Respondents, n (%)
**Assigned mobile app**		
	Kwit	58 (50)
	Quit Genius	58 (50)
**Age (years)**		
	18-29	49 (42.2)
	30-41	41 (35.3)
	42-53	15 (12.9)
	54-65	11 (9.5)
**Gender**		
	Male	71 (61.2)
	Female	45 (38.8)
**Education**		
	Low (primary school)	8 (6.9)
	Medium (secondary school)	21 (18.1)
	High (university or college degree)	87 (75)
**Marital status**		
	Single	77 (66.4)
	Married or civil partnered	39 (33.6)
**Employment status**		
	Employed	76 (65.6)
	Nonemployed	31 (26.7)
	Unemployed	6 (5.2)
	Prefer not to answer	3 (2.6)
**World Health Organization regions**		
	Western Pacific	4 (3.4)
	Americas	10 (8.6)
	Southeast Asia	16 (13.8)
	Europe	67 (57.8)
	Africa	17 (14.7)
	Eastern Mediterranean	2 (1.7)
**Daily smoking (number of cigarettes)**		
	10 or less	63 (54.3)
	11-20	43 (37.1)
	21-30	8 (6.9)
	31 or more	2 (1.7)
**Fagerström nicotine dependence**		
	Low (0-4)	62 (53.4)
	Moderate (5-7)	45 (38.8)
	High (8-10)	9 (7.8)

### Self-Efficacy and Motivation to Quit

**Figure 1:**
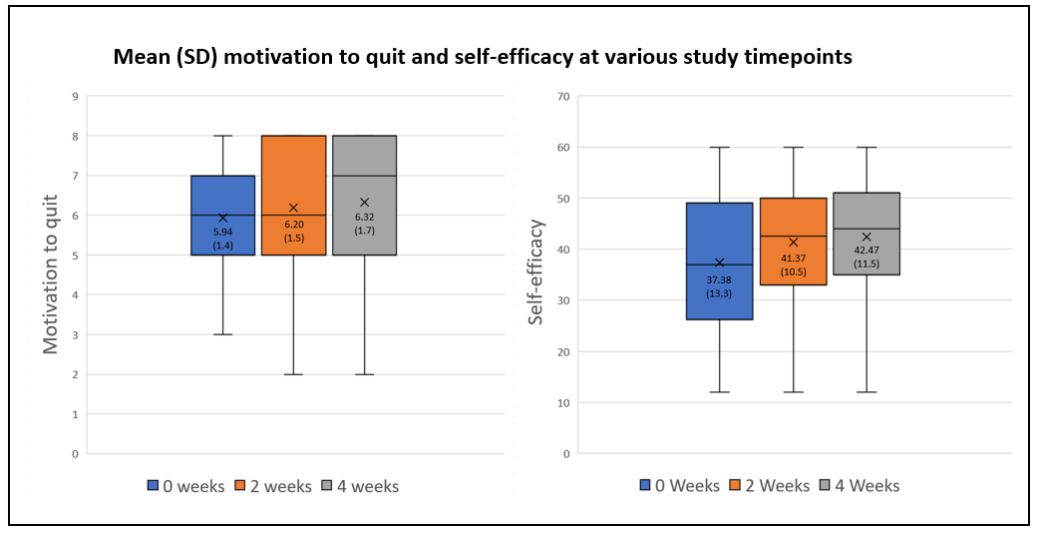
Self-efficacy and motivation to quit at baseline, midstudy, and end-study (n=116).

Figure 1 shows that the mean motivation to quit increased from 5.94 at baseline to 6.20 after 2 weeks of app use and to 6.32 points after 4 weeks of app use. The median motivation to quit (and IQR) at baseline was 6.00 (IQR 5-7), 6.00 (IQR 5-8) at 2 weeks, and 7.00 (IQR 5-8) at 4 weeks. Similarly, the mean self-efficacy score increased from 37.38 points at baseline to 41.37 points after 2 weeks of using the app and then to 42.47 points after 4 weeks of using the app. Median self-efficacy (and IQR) at baseline was 37.00 (IQR 27-39), which increased to 42.50 points (IQR 33-50) at 2 weeks and increased further to 44.00 (IQR 35-51) at 4 weeks. Paired *t* tests found that increases in mean self-efficacy and motivation to quit between baseline and midstudy and baseline and end-study were statistically significant (Multimedia Appendix 4).

### Gamification

Table 2 displays the average midstudy and end-study perceived usefulness, ease of use, and frequency of use of overall gamification and specific gamification features embedded in the apps. At midstudy and end-study, goal setting was perceived to be the most useful gamification feature (4.14 score out of 5), whereas sharing was perceived to be the least useful feature (3.72 at midstudy and 3.28 at end-study out of 5). Participants also perceived goal setting to be the easiest to use feature at both midstudy and end-study (4.31 and 4.36, respectively). In terms of frequency of use, participants self-reported that they used the progress dashboards the most often during both the midstudy and end-study assessments (3.23 and 3.30, respectively). The feature reported to be used the least frequently was the sharing feature, as not many participants shared their progress or results with others.

The linear regression model results presented in Table 3 show that a 1-point increase in average perceived frequency of gamification features was statistically significantly associated with a 3.35-point increase in self-efficacy from baseline to end-study (*β*=3.35; 95% CI 0.31-6.40). The average perceived ease of use and usefulness of gamification were not associated with changes in self-efficacy. In addition, a 1-point increase in baseline self-efficacy was associated with a 1.06-point decrease in self-efficacy between baseline and end-study (*β*=−1.06; 95% CI −1.22 to −0.90). Gender, marital status, nicotine dependence, baseline motivation, average perceived ease of use, and usefulness of gamification features were not statistically significantly associated with changes in self-efficacy from baseline to end-study. The second linear regression model presented in Table 3 shows that individuals with medium or high education had, on average, a 1.31 point (95% CI −2.60 to −0.01) and 1.21 point (95% CI −2.32 to −0.10) lower motivation to quit than individuals with a low level of education. Moreover, a 1-point increase in average perceived frequency of use of gamification features was statistically significantly associated with a 0.54-point increase in motivation to quit at end-study compared with baseline (*β*=.54; 95% CI 0.15-0.94). Similarly, there was some indication that the average usefulness of gamification and baseline self-efficacy are associated with change in motivation to quit. Finally, a 1-point increase in baseline motivation to quit was statistically significantly associated with a 0.69-point decrease in motivation to quit (*β*=−.69; 95% CI −0.90 to −0.49).

**Table 2:** Overview of perceived frequency of use, ease of use, and usefulness of gamification features embedded in the Kwit and Quit Genius apps (n=116).

Gamification features	Midstudy, mean (SD)				End-study, mean (SD)		
	Perceived usefulness^a^	Perceived ease of use^a^	Perceived frequency of use^a^	Perceived usefulness^a^		Perceived ease of use^a^	Perceived frequency of use^a^
Logging diaries	3.78 (0.99)	4.08 (0.95)	3.13 (1.21)	3.85 (0.98)		3.85 (0.97)	3.19 (1.20)
Achievements and badges	3.64 (1.11)	3.91 (0.96)	2.90 (1.27)	3.78 (1.06)		3.97 (1.07)	2.96 (1.23)
Progress tracking	3.91 (0.94)	4.07 (0.86)	3.23 (1.11)	4.04 (0.93)		4.07 (0.96)	3.30 (1.21)
Unlocking levels or competing stages	3.93 (0.89)	4.01 (0.94)	3.03 (1.02)	3.94 (0.93)		4.18 (0.79)	3.09 (1.08)
Sharing feature	3.08 (1.15)	3.72 (0.87)	1.86 (1.13)	3.28 (1.17)		3.72 (0.95)	1.93 (1.16)
Motivation cards^b^	3.64 (0.95)	4.10 (0.79)	2.95 (1.26)	3.71 (1.12)		4.08 (0.98)	3.14 (1.23)
Goal setting^c^	4.14 (0.85)	4.31 (0.71)	2.64 (0.91)	4.14 (0.80)		4.36 (0.81)	2.97 (1.03)
Overall	3.71 (0.75)	4.00 (0.64)	2.83 (0.80)	3.80 (0.78)		4.04 (0.72)	2.92 (0.87)

^a^Range: 1-5.

^b^Only applicable to Kwit.

^c^Only applicable to Quit Genius.

**Table 3:** Linear regression model examining the association between perceived usefulness, ease of use, and frequency of use of gamification features with change in self-efficacy and change in motivation to quit (n=116).

Variables		Change in self-efficacy		Change in motivation to quit	
		*β*	95% CI	*β*	95% CI
Age (years)		−.05	−0.26 to 0.17	−.01	−0.04 to 0.02
**Gender**					
	Male (referent)	Reference	Reference	Reference	Reference
	Female	1.89	−2.48 to 6.26	.19	−0.37 to 0.76
**Nicotine dependence**					
	Low (referent)	Reference	Reference	Reference	Reference
	Moderate	−1.02	−5.50 to 3.46	−.08	−0.66 to 0.50
	High	5.93	−2.15 to 14.02	.42	−0.61 to 1.46
**Education**					
	Low (referent)	Reference	Reference	Reference	Reference
	Medium	−4.95	−15.01 to 5.16	−1.31^a^	−2.60 to −0.01
	High	−8.01	−16.63 to 0.61	−1.21^a^	−2.32 to −0.10
**Marital status**					
	Single (referent)	Reference	Reference	Reference	Reference
	Married	−.10	−5.35 to 5.17	−.03	−0.70 to 0.65
Mean frequency of gamification use		3.35^a^	0.31 to 6.40	.54^a^	0.15 to 0.94
Mean ease of use of gamification		−1.21	−5.16 to 2.74	−.03	−0.54 to 0.48
Mean usefulness of gamification		1.63	−2.53 to 5.79	.51	−0.03 to 1.04
Baseline self-efficacy		−1.06^a^	−1.22 to −0.90	−.02	−0.04 to −0.00
Baseline motivation to quit		1.14	−0.44 to 2.71	−.69^a^	−0.90 to −0.49
Constant		35.79^a^	15.68 to 53.89	3.19	0.73 to 5.65

^a^*P*<.05.

## Discussion

### Principal Findings

We found that the use of Kwit and Quit Genius was associated with increased self-efficacy and motivation to quit levels 4 weeks after app use compared with baseline. Our study also found that the perceived frequency of use of gamification features was associated with an increase in self-efficacy and motivation to quit. Finally, higher baseline self-efficacy and motivation to quit were both associated with smaller increases in self-efficacy and motivation to quit levels 4 weeks after using the mobile apps compared with preapp use.

The key finding from our analyses showed that the frequency of gamification use was associated with increased levels of self-efficacy and motivation to quit after app use compared with before app use. One possible reason for this could be that the frequency of gamification use has an effect on the overall user engagement with the app, which in turn influences the self-efficacy and motivation to quit levels. Some studies in the existing literature have found positive effects of gamification on user engagement. For example, Othman et al [45] found that gamification had a positive impact on user engagement with Play4fit, a fitness smartphone app. Similarly, Looyestyn et al [46] found that gamification was effective in increasing engagement levels with app-based programs. Therefore, higher overall app engagement as a result of gamification could have increased smokers’ confidence in their ability to quit and the level of motivation to quit. Moreover, as higher engagement has been found to be positively associated with intervention effectiveness [47,48], it is possible that apps with gamification features that influence the overall engagement levels are associated with better cessation outcomes than those without gamification features.

Although not explicitly investigated in our study, it is also possible that the frequency of gamification use had an effect on user enjoyment, which in turn affected the motivation to quit and self-efficacy levels. Higher levels of user engagement could intrinsically influence motivation levels, as the use of the app could be rewarding or enjoyable for the user regardless of the final outcome. The theory of flow suggests that people can experience the state of *flow* in which they are highly involved in an activity because it is so enjoyable that they would engage in it even at a cost [49]. Research shows that gamification elements can enhance the level of enjoyment experienced, leading to higher levels of motivation [49]. Another possible theory-based explanation for why the frequency of gamification use was found to be associated with increased self-efficacy levels could be that gamification may influence a user’s competence and confidence. Certain gamification features such as providing immediate feedback on performance, incremental levels, and providing badges of achievement could have provided a low risk way to attempt a task while also increasing confidence levels that their set goals are attainable [50]. According to the self-determination theory, the fulfillment of 3 types of psychological needs (autonomy, competence, and relatedness) can foster motivation [51]. By providing immediate feedback on performance through game elements such as badges, level advancements, and progress tracking, gamification may help fulfill competence needs and enhance self-efficacy. On the other hand, elements such as the sharing feature could help support and enhance the feeling of relatedness and in turn boost motivation levels.

The association between perceived frequency of use of gamification features and increases in motivation to quit and self-efficacy can have important implications for the use of gamification and game design principles in nongame contexts such as health behavior change. We found progress tracking to be the most frequently used gamification feature after 4 weeks of app use. According to a review of smoking cessation mobile apps, this feature was found to be most commonly integrated into apps by app developers [52]. However, we also found that one of the gamification features that users interacted with frequently (unlocking levels or completing stages) was also the feature that was adopted by only 20% of the smoking cessation apps investigated in the review. It could be valuable for app developers to investigate the impact of such gamification features as they are not often integrated into mobile apps but could have the potential to improve user engagement and thereby self-efficacy and motivation to quit. This also highlights the importance and need for collaboration between mobile app developers, researchers, and behavior change specialists to create interventions that can effectively target and influence vital cognitive factors via strategies such as gamification.

Our study also found some indications that the average perceived usefulness of gamification was associated with increased levels of self-efficacy and motivation to quit after 4 weeks of app use compared with baseline. This finding is in accordance with previous studies that have explored the impact of gamification on motivation to quit. For example, Pløhn and Aalberg [26] found that participants who managed to quit smoking after using a gamified app-based cessation program reported the effectiveness of gamification as a motivational factor [26]. Similarly, a study of 16 participants found that individuals who engaged in a gamified cessation intervention had higher levels of motivation than those who engaged with a nongamified cessation intervention [27]. Our findings, supported by other studies, highlight the value of further investigating the usefulness of specific gamification features to better design mHealth solutions geared toward facilitating health behavior change.

In addition to our findings on gamification, a general finding of our analyses was the increase in self-efficacy between baseline and 4 weeks after app use. This implies that participants experienced increased levels of confidence to refrain from smoking not only when faced with both internal stimuli such as cravings and emotions but also when faced with external stimuli such as being surrounded by other smokers or social situations that trigger smoking cravings. Likewise, the increase in motivation to quit between baseline and end-study suggests that participants experienced higher determination to quit and placed greater importance in successfully quitting on the current quit attempt. The association between high self-efficacy and motivation to quit with better cessation outcomes has been established in a large number of previous studies [6-9]. Although we did not assess quit outcomes in our study, the evidence that increased self-efficacy and motivation to quit can lead to better quitting rates is an encouraging finding for gamified smoking cessation apps. Such apps could be considered as possible cessation interventions available to smokers seeking to quit. Increased use of mobile apps for smoking cessation could also have wide-reaching consequences for alleviating health inequalities, as mHealth solutions are able to reach a large number of people at low cost [15].

It is important to note that the majority of increases in both self-efficacy and motivation to quit are evident during the first 2 weeks of app use. This could imply that the gamified mobile apps have a saturated effect after an initial period of using the app, after which they help participants maintain their self-efficacy and motivation levels. Past research has found that an increase in self-efficacy during the course of an intervention can lead to greater likelihood of long-term success [53]. Another study showed that participants who experienced an increase in self-efficacy during the first 2 weeks of a 12-week smoking cessation intervention were significantly more likely to stay quit after treatment [54]. The study also sheds light on the importance of promoting a smoker’s early sense of confidence in their ability to quit to increase the odds of successfully quitting long after the intervention is completed [54]. These findings could have possible implications for future smoking cessation interventions to adopt strategies to raise self-efficacy and motivation levels over the course of the intervention, especially *early on*.

Finally, our analyses also showed that having higher education, baseline self-efficacy, and motivation to quit were associated with smaller improvements in self-efficacy and motivation to quit. This suggests that the gamified mobile apps in our study have a greater benefit for individuals with lower levels of confidence in their ability to quit, individuals with lower determination to quit, and individuals with lower education levels or individuals with lower socioeconomic status. As socioeconomic differences are present in both smoking prevalence and successful cessation, this finding could be used to inform future interventions to help disadvantaged groups and thereby reduce inequalities.

### Limitations

By examining the impact of gamified smoking cessation mobile apps quantitatively, we attempt to address a gap in the existing literature. However, to do so, we developed a questionnaire to quantitatively assess gamification that requires participant self-report. Self-reporting may have led to different or inaccurate perceptions, particularly when answering questions regarding frequency of use. Moreover, the developed questionnaire has not yet been scientifically validated. Future research could test the validity and reliability of the questionnaire developed to assess gamification more rigorously. Another drawback of our research was that the majority of participants reported having a low to moderate dependence on nicotine. It could be that the findings differed among individuals with high nicotine dependence. Therefore, future research could explore the differences between the various types of smokers. Similarly, participants with mental health conditions were not eligible to participate in the study, and it could be that the findings are not generalizable to all members of the population. Finally, our study comprised motivated volunteers, which could subject the findings to volunteer bias.

To address the natural limitations of this study design, future research could consider running randomized controlled trials with 2 smoking cessation apps that are as similar as possible, differing only in the number of gamification elements or, more importantly, the type of gamification elements to robustly test the impact of gamification. Our study was underpowered to investigate the impact of individual game elements; therefore, we were unable to explore the effects of and differences between game elements. Future studies could try to isolate and test individual game elements within behavior change interventions, as not all gamification elements may have the same impact or function in the same way. It could be that certain gamification elements interact with other elements or with individual dispositions, situational circumstances, and the characteristics of particular target activities differently than others [20]. Consequently, it is vital for future research to focus on theory-driven studies that investigate how individual gamification elements work.

### Conclusions

In conclusion, our research found that more frequent engagement with gamification features in smoking cessation apps was associated with higher self-efficacy and motivation to quit. The findings of this study provide a good platform for further investigation into the role of gamification in improving important cognitive factors essential for the quitting process of smokers. Future studies should continue to explore the impact and usefulness of gamification in the context of mHealth. On the basis of our findings and existing literature, we recommend that mobile app developers collaborate with behavior change specialists to develop more tailored, evidence-based, and theory-driven interventions. At the same time, app developers should be encouraged to work together with scientists to explore and test strategies, such as gamification, that could target vital psychological components of behavior change while possibly improving engagement with the app.

## Appendix

Multimedia Appendix 1 Study participant flowchart.

Multimedia Appendix 2 Screenshots of Quit Genius and Kwit.

Multimedia Appendix 3 Gamification features and tactics by Cugelman embedded in the Kwit and Quit Genius apps.

Multimedia Appendix 4 The t tests statistically examining the mean differences in self-efficacy and motivation to quit scores between study time points (n=116).

## Abbreviations

CBT: cognitive behavioral therapy
ICREC: Imperial College London Research Ethics Committee
mHealth: mobile health
PID: participant identification number
