# Supplementary figures and images for: Impact of Gamification on the Self-Efficacy and Motivation to Quit of Smokers: Observational Study of Two Gamified Smoking Cessation Mobile Apps
Source: JMIR Serious Games. 2021 Apr 27;9(2):e27290. doi: 10.2196/27290 (PMC8114162; doi:10.2196/27290)

## Supplementary File: Figure 1 (APPENDIX 1)

**
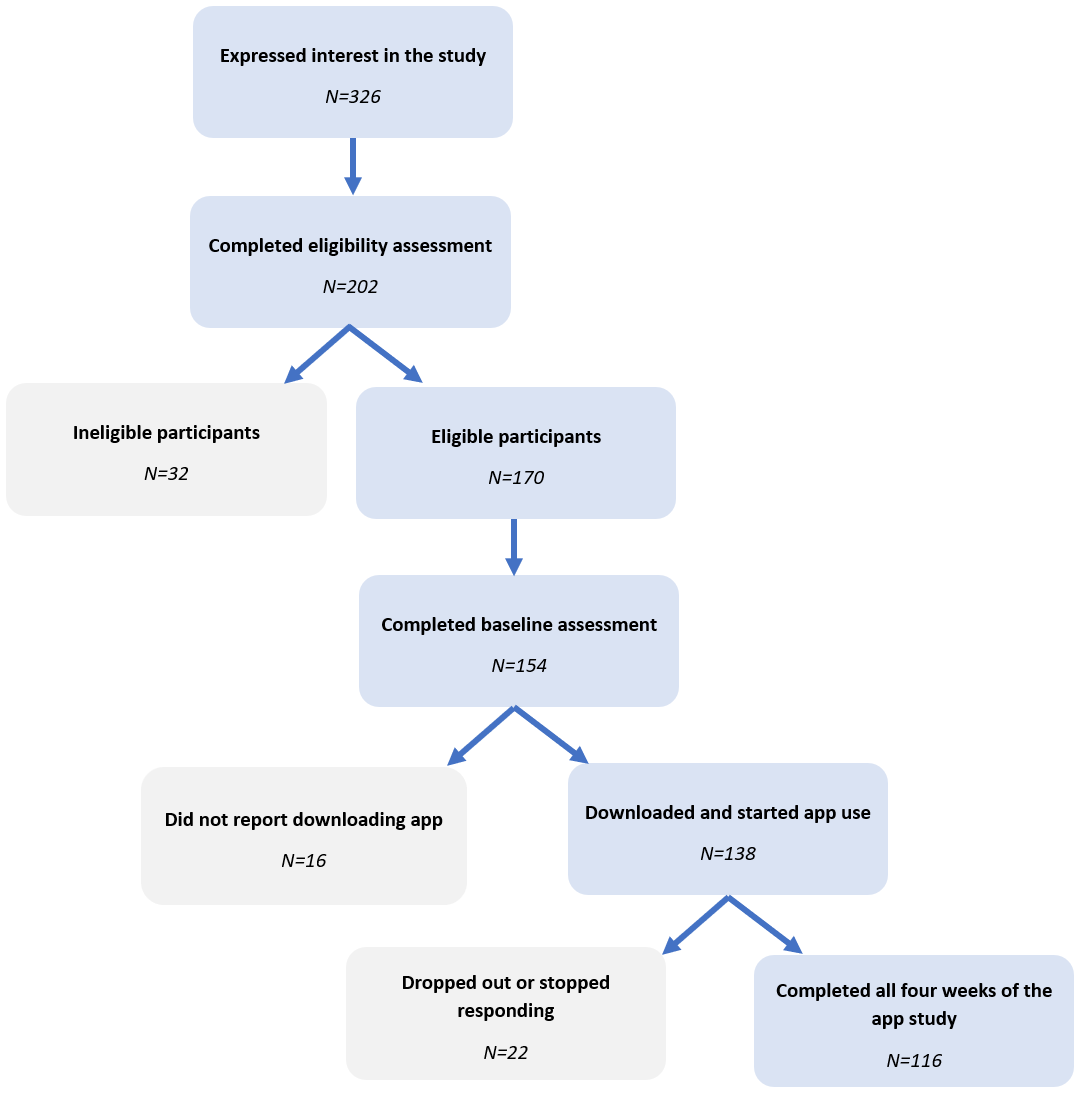
**Supplementary Figure 1. Study Participants Flowchart

Supplement: Multimedia Appendix 1 [file games_v9i2e27290_app1.docx]
