# Supplementary material for: Impact of Gamification on the Self-Efficacy and Motivation to Quit of Smokers: Observational Study of Two Gamified Smoking Cessation Mobile Apps
Source: JMIR Serious Games. 2021 Apr 27;9(2):e27290. doi: 10.2196/27290 (PMC8114162; doi:10.2196/27290)
Supplement: Multimedia Appendix 2 [file games_v9i2e27290_app2.docx]

## Supplementary File: Screenshots of Mobile Apps (APPENDIX 2)


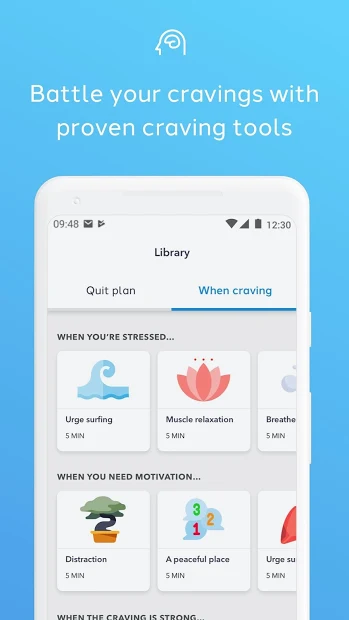

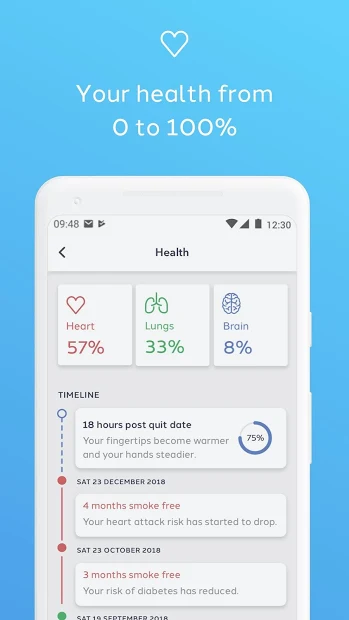

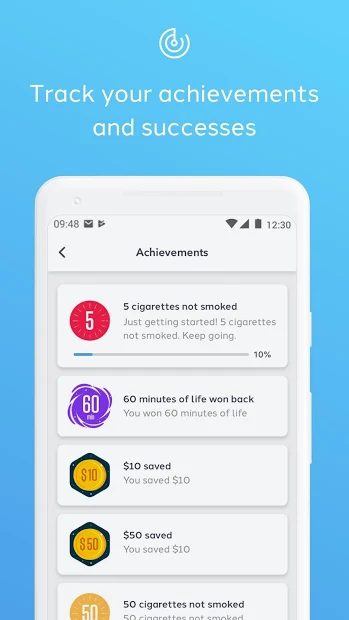

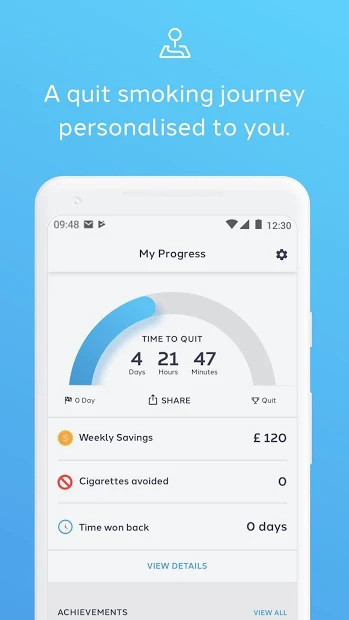
1. Screenshots of Quit Genius


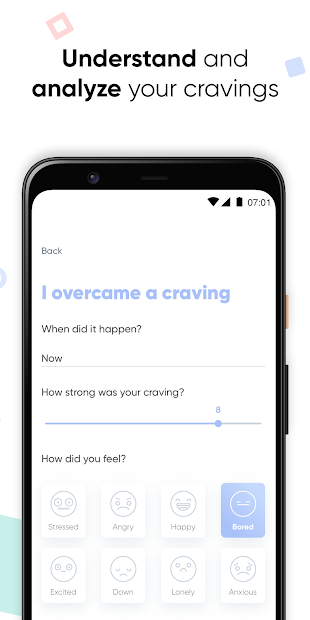

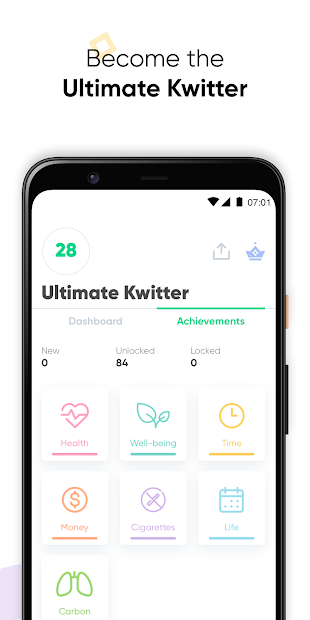

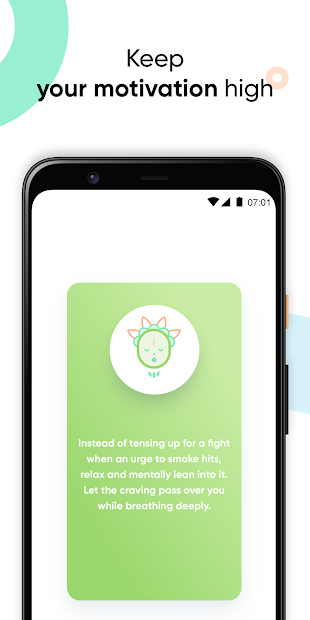

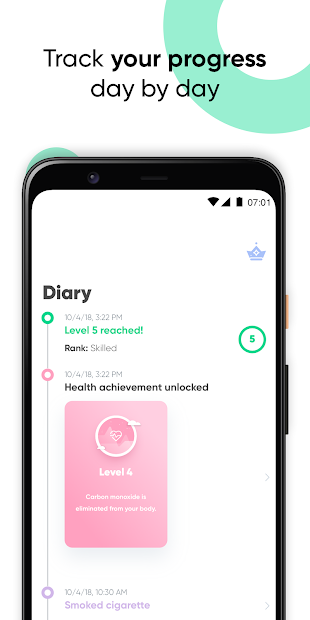
2. Screenshots of Kwit
