# Supplementary material for: Impact of Gamification on the Self-Efficacy and Motivation to Quit of Smokers: Observational Study of Two Gamified Smoking Cessation Mobile Apps
Source: JMIR Serious Games. 2021 Apr 27;9(2):e27290. doi: 10.2196/27290 (PMC8114162; doi:10.2196/27290)
Supplement: Multimedia Appendix 3 [file games_v9i2e27290_app3.docx]

## Supplementary File: Table 1 (APPENDIX 3)

Supplementary Table 1. Cugelman’s (2013) Gamification Features and Tactics Embedded in the apps Kwit and Quit Genius

| **Gamification Strategy/Tactic** | **Kwit** | **Quit Genius** |
| --- | --- | --- |
| Capacity to overcome challenges, learning and development | Smoking diaries; Motivation cards | Smoking diaries |
| Reinforcement; giving rewards, providing badges for achievements | Achievements with pop-up feedback | Badges and trophies with pop-up feedback |
| Providing feedback on performance; showing progress | Progress dashboard to track money saved, benefits accrued | Progress dashboard to track money saved, benefits accrued |
| Using levels or incremental challenges | Unlocking levels | Incremental stages |
| Compare progress with self and others | Share on social media, texting, e-mail etc. feature | Share on social media, texting, e-mail etc. feature |
| Goal setting; providing clear goals | N/A | Setting a quit date/goal |
