# Supplementary material for: Impact of Gamification on the Self-Efficacy and Motivation to Quit of Smokers: Observational Study of Two Gamified Smoking Cessation Mobile Apps
Source: JMIR Serious Games. 2021 Apr 27;9(2):e27290. doi: 10.2196/27290 (PMC8114162; doi:10.2196/27290)
Supplement: Multimedia Appendix 4 [file games_v9i2e27290_app4.docx]

## Supplementary File: Table 1 (APPENDIX 4)

Supplementary Table 1. T-Tests Statistically Examining Mean Differences in Self-Efficacy and Motivation to Quit Scores Between Study Timepoints (N=116)

|  | **Baseline vs. Mid-Study** | **Baseline vs. End-Study** | **Mid-Study vs. End-Study** |
| --- | --- | --- | --- |
| **Self-Efficacy**  **(12 to 60)** | 3.99*  (1.16 to 6.82) | 5.09*  (1.83 to 8.34) | 1.09  (-1.02 to 3.21) |
| **Motivation to Quit**  **(2 to 8)** | 0.26*  (0.01 to 0.51) | 0.38*  (0.06 to 0.70) | 0.12  (-0.10 to 0.34) |

*P-value < 0.05
